# Gender and Racial Profile of the Academic Pediatric Faculty Workforce in the United States

**DOI:** 10.7759/cureus.22518

**Published:** 2022-02-23

**Authors:** Sundas Saboor, Sadiq Naveed, Amna M Chaudhary, Munira Jamali, Mehwish Hussain, Javed Siddiqi, Faisal Khosa

**Affiliations:** 1 Department of Public Health, Harvard T.H. Chan School of Public Health, Boston, USA; 2 Psychiatry, Hartford Hospital - Institute of Living, Hartford, USA; 3 Department of Psychiatry, University Hospitals/Case Western Reserve University, Ohio, USA; 4 Department of Internal Medicine, Dow Medical College, Dow University of Health Sciences, Karachi, PAK; 5 Department of Public Health, Imam Abdurrahman Bin Faisal University, Dammam, SAU; 6 Department of Neurosurgery, Desert Regional Medical Center, Palm Springs, USA; 7 Department of Neurosurgery, Riverside University Health System Medical Center, Moreno Valley, USA; 8 Department of Neurosurgery, Arrowhead Regional Medical Center, Colton, USA; 9 Department of Neurosurgery, California University of Science and Medicine, Colton, USA; 10 Department of Radiology, Vancouver General Hospital, Vancouver, CAN

**Keywords:** race, gender, pediatrics, academic faculty, disparity

## Abstract

Background

Equity, diversity, and inclusion remain a challenge in the healthcare workforce. This study explored the current gender and racial/ethnic trends in academic pediatric positions across the United States.

Methodology

The pediatric faculty self-reported data by the American Association of Medical Colleges (AAMC) Faculty Roster from 2007 to 2020 were analyzed. The races were classified as White (non-Hispanic), Asian, Hispanic, Black (non-Hispanic), Multiple races (including both non-Hispanic and Hispanic), Others, and Unknown. Gender was categorized as male and female.

Results

The results showed that Asian, Black (non-Hispanic), and Hispanic academic pediatricians increased in full professor, associate professor, and assistant professor positions and decreased in instructor positions from 2007 to 2020. Black (non-Hispanic) academic pediatricians relatively decreased 5.5% in chairperson positions. Women increased in full professor, associate professor, instructor, and chairperson positions; however, relatively decreased 1.8% in assistant professor positions. Men and White (non-Hispanic) academic pediatricians relatively decreased 10.5% and 16%, respectively, in all academic ranks. Women, Asian, Black (non-Hispanic), Hispanic, and Other races were underrepresented in tenured, on-track (tenure-eligible), and not-on-track (tenure-eligible) positions.

Conclusions

Women and underrepresented minorities in medicine (URiM) physicians continue to remain significantly underrepresented in academic pediatric faculty positions and tenured track positions. There is a dire need to adapt multifaceted strategies to increase the engagement of women and URiM in academic pediatrics.

## Introduction

Diversity among healthcare professionals is vital to optimally serve diverse patients and ensure the provision of quality healthcare [1]. Minority physicians ensure to provide culturally sensitive care to ethnically concordant patients and serve as mentors to minority medical students. Despite the recent augmented focus on increasing diversity, equity in academic medicine remains an elusive dream [2].

The American Association of Medical Colleges (AAMC) defines underrepresented minorities in medicine (URiM) as those who belong to racial populations that are considerably lower in medicine than their numerical representation in the general population. Previous studies examining races and gender of undergraduate, graduate, and faculty positions reported only modest improvement in URiM proportions within medicine over a large period. The representation quotient (RQ) (the ratio of the proportion of a subgroup among the total population of applicants/matriculants relative to the corresponding estimated proportion of that subgroup in the US population) has been defined for all races by Lett et al. [3]. From 2002 to 2017, the RQ for Black (non-Hispanic) female applicants declined from 0.75 to 0.66, Hispanic female applicants varied between 0.4 and 0.47, while the RQ for American Indian/Alaskan Native applicants was 0.35. Between 2002 and 2017, Black (non-Hispanic), Hispanic, Asian/Pacific Islander, Native American/Alaskan, and women medical school applicants and matriculates were underrepresented, with a downward trend for Black (non-Hispanic) and Asian/Pacific Islander female applicants [3]. Generally, all URiM faculty in US medical schools has increased from 6.8% in 2000 to 8% in 2010; however, their promotion to seniority ranks lags in all medical disciplines [4,5]. In academic pediatrics in 2012, according to the AAMC, there were 0.15% American Indian/Alaskan Native, 3.3% Black/African American, 4.7% Hispanic, and 11% Asian academic pediatricians compared to 63% White academic pediatricians [6]. This disparity for the URiM pediatric faculty compared to the White (Non-Hispanic) pediatric faculty is also evident in other studies [7,8].

Racial disparities are prominent in pediatric residency programs. From 2007 to 2019, trends for URiM remained unchanged for pediatric residents (16% in 2007 to 16.5% in 2019), while overall there was a downward trend for pediatric fellows (14.2% in 2007 to 13.5% in 2019) [9]. Furthermore, disparities have also been observed in clinical training, medical faculty appointments, and journal editorial boards of other medical specialties [10,11]. Lower probability of receiving National Institutes of Health (NIH) investigator-initiated research funding, less likelihood of receiving mentored NIH awards, negotiating for protected research time, racism and discrimination, coping with isolation as an URiM faculty, and inadequate mentorship are possible deterrents for minorities to succeed in academia [12].

Women are also underrepresented in medicine [13]. Although the total number of female medical school matriculants has surpassed male medical school matriculants in recent years, this parity has not translated in the proportion of female faculty [14]. Recent studies have suggested that only 21% of full professors and 15% of departmental chairs are women, while in pediatrics only 40% of senior faculty positions and 20% of department chairs are women. Fewer opportunities for academic growth and a dearth of women leadership role models are some of the reasons for the lack of diversity in academic medicine [15].

Medical organizations such as the AAMC and American Medical Association (AMA) are making efforts to diversify medicine. The main purpose of their agenda is to enable programs to implement policies to recruit more URiM in medicine to satisfy sponsoring institutions’ missions [16]. As the pediatric workforce has shown a lack of diversity in recent years, a close examination of the racial and gender gap in academic pediatrics is warranted. This study analyzes the gender and racial trends in academic pediatrics in the United States over 14 years (2007-2020).

## Materials and methods

This retrospective cross-sectional analysis presents data from the pediatric faculty across the United States from 2007 to 2018. Data were obtained from the AAMC Faculty Roster Data [17]. Tenure track data were obtained from 2007 to 2020. Institutional Review Board consent was not required for this study as no humans or animals were involved.

Variables

The AAMC report includes information on the department, academic rank, gender, race/ethnicity, and tenure of full-time faculty. Race/ethnicity was categorized as self-reported White (non-Hispanic), Asian, Hispanic, Black (non-Hispanic), Mixed races (both non-Hispanic and Hispanic), Others (American Indians, Alaskan Natives, Native Hawaiian, other Pacific Islanders, others), and Unknown. Gender was categorized as self-reported male and female. Academic rank was categorized as a full professor, associate professor, assistant professor, instructor, other, and chairperson. Tenure classification was categorized as tenured, on track (tenure-eligible), not on track (tenure-eligible), tenure not available, and missing data.

Gender and racial distribution by year and across academic ranks are described. Counts and percentages were calculated to highlight trends in faculty appointments over 14 years and across ranks.

## Results

Distribution of all academic pediatricians in the United States from 2007 to 2020

There was a relative increase of 37.7% of all academic pediatricians in the United States from 2007 to 2020 (Table 1). Between 2007 and 2020, male academic pediatricians relatively decreased 10.5%, while female academic pediatricians increased 11.1%. Asian, Black (non-Hispanic), and Hispanic academic pediatricians relatively increased 50%, 20%, and 24.3%, respectively. White academic pediatricians decreased 16% from 2007 to 2020.

**Table 1:** Absolute and relative percentage change in academic pediatricians between 2007 and 2020 by gender and race. Others include American Indians, Alaskan Natives, Native Hawaiian, Pacific Islanders, etc. “+” denotes an increase, and “-” denotes a decrease.

Positions	2007, % (n)	2020, % (n)	% Absolute change	% Relative change
All academic pediatricians	100 (15,409)	100 (21,226)		+37.7
Gender				
Male	51.4 (7,919)	46.0 (9,754)	-5.4	-10.5
Female	48.6 (7,490)	54.0 (11,472)	+5.4	+11.1
Race				
White (non-Hispanic)	71.5 (11,011)	60.0 (12,721)	-11.5	-16.0
Asian	13.4 (2,063)	20.1 (4,278)	+6.7	+50.0
Black (non-Hispanic)	4.0 (623)	4.8 (1,032)	+0.8	+20.0
Hispanic	3.7 (566)	4.6 (972)	+0.9	+24.3
Multiple Race	3.7 (577)	5.6 (1,195)	+1.9	+51.4
Unknown	2.8 (433)	3.3 (710)	+0.5	+17.9
Others	0.9 (136)	1.5 (318)	+0.6	+66.7
Full professors	100 (3,118)	100 (4,282)		37.3
Gender				
Male	72.7 (2,267)	60.9 (2,612)	-11.8	-16.2
Female	27.3 (851)	39.0 (1,670)	+11.7	+42.9
Race				
White (non-Hispanic)	84.7 (2,642)	77.5 (3,320)	-7.2	-8.5
Asian	6.7 (209)	11.8 (506)	+5.1	+76.1
Black (non-Hispanic)	1.5 (47)	2.4 (104)	+0.9	+60.0
Hispanic	2.6 (80)	3.1 (133)	+0.5	+19.2
Multiple Race	2.9 (89)	3.8 (163)	+0.9	+31.0
Unknown	1.3 (40)	0.9 (40)	-0.4	-30.8
Others	0.4 (11)	0.4 (16)	0.0	0.0
Associate professors	100 (3,200)	100 (5,193)		+62.3
Gender				
Male	58.2 (1,861)	47.1 (2,447)	-11.1	-19.1
Female	41.8 (1,339)	52.8 (2,746)	+11.0	+26.3
Race				
White (non-Hispanic)	77.9 (2,494)	67.0 (3,483)	-10.9	-14.0
Asian	10.8 (345)	18.5 (961)	+7.7	+71.3
Black (non-Hispanic)	3.2 (101)	3.8 (197)	+0.6	+18.8
Hispanic	3.1 (99)	3.8 (196)	+0.7	+22.6
Multiple Race	3.2 (101)	4.6 (237)	+1.4	+43.8
Unknown	1.5 (49)	1.3 (68)	-0.2	-13.3
Others	0.3 (11)	0.9 (51)	+0.6	+200.0
Assistant professors	100 (6,955)	100 (8,701)		+25.1
Gender				
Male	44.3 (3,080)	45.3 (3,942)	+1.0	+2.3
Female	55.7 (3,875)	54.7 (4,759)	-1.0	-1.8
Race				
White (non-Hispanic)	64.6 (4,494)	46.3 (4,026)	-18.3	-28.3
Asian/Pacific Islander	17.1 (1,187)	27.9 (2,426)	+10.8	+63.2
Black (non-Hispanic)	5.4 (375)	7.1 (622)	+1.7	+31.5
Hispanic	4.4 (305)	6.0 (519)	+1.6	+36.4
Multiple Race	4.6 (318)	7.7 (668)	+3.1	+67.4
Unknown Race	2.8 (197)	3.2 (276)	+0.4	+14.3
Others	1.1 (79)	1.9 (164)	+0.8	+72.7
Instructors	100 (1,850)	100 (2,453)		+32.6
Gender				
Male	31.2 (577)	22.6 (555)	-8.6	-27.6
Female	68.8 (1,273)	77.4 (1,898)	+8.6	+12.5
Race				
White (non-Hispanic)	65.0 (1,203)	61.6 (1,511)	-3.4	-5.2
Asian	15.2 (282)	12.7 (312)	-2.5	-16.4
Black (non-Hispanic)	4.9 (90)	3.2 (79)	-1.7	-34.7
Hispanic	4.0 (74)	3.8 (92)	-0.2	-5.0
Multiple Race	2.8 (51)	4.2 (104)	+1.4	+50.0
Unknown	6.3 (116)	11.0 (271)	+4.7	+74.6
Others	1.8 (34)	3.4 (84)	+1.6	+88.9
Other positions	100 (286)	100 (443)		+54.9
Gender				
Male	37.1 (106)	20.8 (92)	-16.3	-44.0
Female	62.9 (180)	79.2 (351)	+16.3	+26.0
Race				
White (non-Hispanic)	62.2 (178)	59.1 (262)	-3.1	-5.0
Asian	14.0 (40)	14.0 (62)	0.0	0.0
Black (non-Hispanic)	3.5 (10)	5.0 (22)	+1.5	+42.9
Hispanic	2.8 (8)	5.4 (24)	+2.6	+92.9
Multiple Race	6.3 (18)	3.8 (17)	-2.5	-39.7
Unknown	10.8 (31)	12.4 (55)	+1.6	+14.8
Others	0.3 (1)	0.2 (1)	-0.1	-33.3
Chairpersons	100 (128)	100 (154)		+20.3
Gender				
Male	82.8 (106)	68.8 (106)	-14.0	-16.9
Female	17.2 (22)	31.2 (48)	+14.0	+81.4
Race				
White (non-Hispanic)	81.3 (104)	77.2 (119)	-4.1	-5.0
Asian	2.3 (3)	7.1 (11)	+4.8	+208.7
Black (non-Hispanic)	5.5 (7)	5.2 (8)	-0.3	-5.5
Hispanic	3.1 (4)	5.2 (8)	+2.1	+67.7
Multiple Race	5.5(7)	3.9 (6)	-1.6	-29.0
Unknown	0.8 (1)	0.0 (0)	-0.8	-100
Others	1.6 (2)	1.3 (2)	-0.3	-18.75

Distribution of race/ethnicity by academic rank from 2007 to 2020

Full pediatric professors increased (+) 37.3% from 2007 to 2020 (Table 1). In the full professor category, Asian academic pediatricians increased 76.1% followed by Black (non-Hispanic) academic pediatricians (+60%) and Hispanic academic pediatricians (+19.2%). White (non-Hispanic) academic pediatricians decreased 6.9% during the same time.

Pediatric associate professors relatively increased 62.3% from 2007 to 2020 (Table 1). Asian academic pediatricians increased 71.3%, Black (non-Hispanic) academic pediatricians increased 18.8%, and Hispanic pediatricians increased 22.6%. White (non-Hispanic)) academic pediatricians relatively decreased 14%.

Pediatric assistant professors increased 25.1% from 2007 to 2020 (Table 1). In the assistant professor category, Asian academic pediatricians increased 63.2%, Black (non-Hispanic) academic pediatricians increased 31.5%, and Hispanic academic pediatricians increased 36.4%. White (non-Hispanic) relatively decreased by 28.3%.

Pediatric instructors increased 32.6% from 2007 to 2020 (Table 1). However, all races decreased relatively in total. Asian, Black (non-Hispanic), Hispanic, and White (non-Hispanic) decreased 16.4%, 34.7%, 5%, and 5.2%, respectively.

In other pediatric faculty positions, there was no relative change noticed for the Asian faculty (Table 1). Black (non-Hispanic) faculty increased 42.9%, while Hispanic increased 92.9%. White (non-Hispanic) faculty relatively decreased 5%.

In chairperson positions, Black (non-Hispanic) faculty relatively decreased 5.5% (Table 1). Asian faculty and Hispanic faculty increased 208.7% and 67.7%, respectively. White (non-Hispanic) faculty relatively decreased 5%. The relative and absolute changes for Multiple, Unknown, and Other racial categories from 2007 to 2020 can be seen in Table 1.

Distribution of gender by academic rank from 2007 to 2020

The data analysis showed that all male academic pediatricians relatively decreased (-) in all academic faculty categories (except the assistant professor category with a relative change of 2.3%) from 2007 to 2020 (Table 1). The relative change for male full professors, associate professors, instructors, other positions, and chairpersons were -16.2%, -19.1%, -27.6%, -44%, and -16.9%, respectively. The relative change for female full professors, associate professors, instructors, other positions, and chairperson were +42.9%, +26.3%, +12.5%, +26% +81.4%, respectively (Table 1). However, female assistant professors in pediatrics relatively decreased 1.8% from 2007 to 2020.

Racial and gender comparison of tenure track

Races and genders differed significantly by tenure track from 2007 to 2020 (Table 2). The 14-year average showed that White (non-Hispanic) academic pediatricians were in majority in each tenure track category, followed by Asian academic pediatricians. The proportion of the White (Non-Hispanic) academic pediatricians was the highest in the category of tenured (41%) while both “on track” (tenure-eligible) and “not on track” (tenure-eligible) were 33%. Overall, 4% Asian faculty represented tenure track, 8% Asian faculty represented on-track (tenure-eligible) and not-on-track (tenure-eligible) ranks (Table 2). Black (Non-Hispanic) and Hispanic faculty represented 1% and 2% in tenured track, respectively; however, both races lagged other tenure academic ranks compared to other races.

**Table 2:** Racial and gender representation of tenured tracks in academic pediatrics (14-year average). Others include American Indians, Alaskan Natives, Native Hawaiian, Pacific Islanders, etc. *: tenure-eligible

	Tenured (%)	On track (%) *	Not on track (%) *	Tenure not available (%)	Missing (%)
Race					
White (non-Hispanic)	41%	33%	33%	34%	31%
Asian	4%	8%	8%	9%	8%
Black (non-Hispanic)	1%	2%	2%	2%	2%
Hispanic	2%	2%	2%	2%	1%
Multiple Race	2%	2%	2%	2%	2%
Unknown	0%	1%	2%	1%	5%
Others	0%	1%	1%	1%	0%
Gender					
Male	34%	23%	21%	21%	21%
Female	16%	27%	29%	29%	29%

The data analysis from 2007 to 2020 (14-year average) showed that male pediatricians represented a majority in the tenured track category (34%). Overall, 23% of male pediatricians were on track (tenure-eligible), while 21% were in the category of “not on track” (tenure-eligible). Female faculty representation increased in categories of “on track” (tenure-eligible) (27%) and “not on track” (tenure-eligible) (29%). However, female faculty lagged in tenured positions (16%) (Table 2).

## Discussion

This study investigated the academic ranks, gender, and racial distribution of pediatric faculty. Despite the significant increase in the total number of academic pediatricians in the last 12 years (2007-2018), significant racial differences persist (Table 1). White (non-Hispanic) pediatricians in academic positions were in majority in total; however, the average White (Non-Hispanic) academic pediatricians decreased from 2007 to 2020. Asian faculty were overrepresented in all academic ranks except the instructor position. The most underrepresented races in our study were Black (non-Hispanics) and Hispanic faculty. Black (non-Hispanic) pediatricians were underrepresented in both high and low-tier academic positions, which is concerning.

Our study found significant discrepancies for URiM in tenure tracks. White (non-Hispanic) academic pediatricians were in majority in all tenured tracks (Table 2). White (non-Hispanic) academic pediatricians were followed by Asian pediatricians. Black (non-Hispanic) and Hispanic academic pediatricians lagged in all tenured tracks. Previous studies also concluded that URiM faculty members were less likely to be promoted to senior ranks despite their increased representation. The inequality for the Hispanic and Black (non-Hispanic) faculty has also been suggested by the AAMC, which concludes that the Hispanic faculty make up only 4% and the Black (Non-Hispanic) faculty make up only 4.2% of the medical faculty [1,18]. 

Likewise, significant trends were noticed while comparing the ranks of men and women in academic pediatrics. Although the total recruited male pediatricians in academic positions were more than female pediatricians, the average number of male academic pediatricians decreased from 2007 to 2020. Overall, female pediatricians increased in faculty appointments from 48.6% in 2007 to 54% in 2020. Significant progress was observed at both low-level and mid-level academic ranks; in fact, female faculty has surpassed male faculty at the level of instructor, associate professor, and full professor positions. However, female faculty were underrepresented in the assistant professor category (-1.8%). For tenured tracks, male pediatric faculty (35%) was overrepresented compared to female pediatric faculty (16%). Our findings are consistent with women’s underrepresentation in higher academic positions previously reported in academic pediatrics [1,15].

As we reflect on the causes of this disparity, the answers are multifactorial. The rise of the Asian faculty can be linked to the rise of the Asian population in the United States in recent years [19]. In the last 20 years, Asians are the fastest-growing ethnic group in the United States, resulting in increased representation in US academic medicine [20]. Asian Americans are frequently considered as an “overrepresented” minority because all Indians, Chinese, Korean, Filipino, Japanese, and Indonesian are categorized as Asians in medical school applications [21]. A survey among Asian/Pacific Islanders in 2013 among Asian medical students reported that an intellectually stimulating learning environment, participating in teaching activities, and securing mentors of the same ethnicities were a few factors that contribute to supporting the inclusion of Asian minorities in academic medicine [22]. Although Asian physicians are now categorized as an overrepresented minority in the United States, Asian faculty still lag in leadership positions in academic medicine [1,19].

On the other hand, Black (non-Hispanics) and Hispanic pediatricians have been underrepresented in both academic positions and tenured tracks. The reason can be attributed to the fact that URiM faculty often have limited opportunities and knowledge regarding the tenure track process. URiM advocating for diversity may feel discouraged to contribute. Due to societal barriers, Blacks (non-Hispanic) are seldom offered research opportunities, which diverts them from meaningful research. Their expertise is not fully recognized by their colleagues, due to which they spend more time in volunteer research work rather than paid academic or clinical work [8]. In one study, 48% of minority academic pediatricians reported inadequate financial resources, 45% reported poor recruitment efforts, 21% reported inadequate opportunities for career advancement, 14% reported fewer resources for research, and 22% reported inadequate research support as a barrier for successful recruitment and retention of minority physicians [23]. All these cumulative factors result in a lesser representation of the Blacks (non-Hispanic) and Hispanics in leadership positions [8]. Barriers faced by URiM faculty also include the phenomenon of “minority tax” and “impostor syndrome.” Minority tax is defined as the burden of extra responsibilities placed on minority faculty to achieve diversity. URiM may be disproportionately charged to lead institutional diversity initiatives. These commitments often come with time allocation or resources and provide less time for scholarly productivity necessary for academic promotion. URiM faculty feel responsible to serve as mentors for URiM students without having adequate mentorship for themselves [24]. Impostor syndrome is characterized by persistent doubt concerning one’s abilities or accomplishments accompanied by the fear of being exposed as a fraud despite evidence of one’s ongoing success. Being a faculty member can be stressful during which well-trained faculty may question their sustainability for academic medicine, even if they are successful [25].

On the other hand, female academic pediatricians have superseded male academic pediatricians in associate, assistant, and instructor positions; however, female academic pediatricians are less likely to attain senior-level positions than male academic pediatricians [26]. The increase of women in academic positions may be attributed to flexible family leaves policies, part-time health benefits for women, and extension of probationary period (tenure track) [27]. In 2011, only 13% of women in academic medicine attained full professor rank, while only 12% of medical school deans and 14% of department chairs were women [28]. The barriers to the promotion of female pediatricians in higher academic positions include low seniority mentorship, fewer authorships, and reduced citations [1]. Other noteworthy factors include differential family responsibilities, workplace harassment, and personal preferences [15]. The slower progress of women in academic medicine can also be attributed to fewer work hours per week, unconscious gender bias, reduced faculty employment rate, and funding opportunities [26].

A diverse faculty is integral to facilitating the recruitment of minority students while fostering an environment conducive to development. Women and URiM faculty are keys to reducing health disparities and serving the current dynamics of the patient population. Minority faculty can help accelerate medical research to train students in providing culturally competent care and leadership that is beneficial for everyone.

Keeping these predictions in perspective, there is a dire need to adapt multifaceted strategies to increase minorities in medicine. Medical schools need to examine the reasons for ethnic disparities, starting from medical school enrollments. Promotion criteria should be reviewed to appreciate the contributions of URiM faculty in education and administration [29]. Some notable initiatives have already been introduced to enhance the representation of URiM in academic pediatrics such as the Academic Pediatric Association’s New Century Scholars Program and the Premedical Honors College and the intensive Summer Medical and Dental Education Program for URiM [1,5]. Investment in these types of programs will be the most effective strategy to improve diversity among faculty and remove the potential barriers faced by marginalized groups in academic pediatrics [5]. Previously, recommendations have been made for policymakers to ensure an adequate supply of diverse physicians and allow the graduate medical education (GME) system to operate in a culturally competent environment. These recommendations include channeling a large percentage of GME funds to academic departments to recruit minority faculty; maintaining financial support for pediatric GME; rejecting unnecessary layers of regulations for minority faculty; and ensuring that the AAMC and ACGME retain their pre-eminent roles in overseeing medical education to serve minorities optimally. GME legislation bill currently pending before Congress includes the Resident Physician Shortage Reduction Act (H.R. 2267/S. 1301) to improve and preserve the GME system to help people from all races. As opportunities for high-impact translational research for women and URiM grow, a diverse physician-scientist workforce will become even more compelling to assist the healthcare system [30]. Lengthening probationary periods, expanding the time allowed to earn tenure, and permitting part-time faculty to obtain tenure can assist URiM and women to pursue academic pediatrics [27].

Limitations

There are several limitations to our study. This study did not explore the superimposed effects of gender and a racial minority, such as a female Hispanic or a male Asian physician, etc. There was insufficient data to consider the representation of various other Asian ethnicities, for example, Filipino, Vietnamese, Korean, Japanese, etc. in detail. Because the AAMC data is cross-sectional, self-reported, and anonymous, we were unable to delve deeper into explanations of the observed results. The data is limited due to the absence of information regarding transgender and non-binary categories in the AAMC data classification. There is no information in the AAMC Faculty Roster regarding age, years of experience, educational track, and institution type (e.g., R1 grants, etc.). Finally, we have some categories such as Other and Unknown that did not seem of any relevance as the exact nature of the positions and racial identity were not recognized.

## Conclusions

Despite an increase in minority appointments over the last decade, disparities continue to prevail in academic ranks in pediatrics. Further research is needed to investigate and mitigate additional factors associated with this misrepresentation. Efforts at all academic ranks and tenures are required to foster immense support for minority faculties to reduce disparities in academic pediatrics. Interventions at recruitment and promotion levels will lead to the diversification of the academic pediatric faculty, ultimately improving patient outcomes in the United States.
